# Evanescent Properties of Optical Diffraction from 2-Dimensional Hexagonal Photonic Crystals and Their Sensor Applications

**DOI:** 10.3390/ma11040549

**Published:** 2018-04-03

**Authors:** Yu-Yang Liao, Yung-Tsan Chen, Chien-Chun Chen, Jian-Jang Huang

**Affiliations:** Graduate Institute of Photonics and Optoelectronics, National Taiwan University, Taipei 1kin6, Taiwan; yuyang12014@gmail.com (Y.-Y.L.); d03941013@ntu.edu.tw (Y.-T.C.); r05941110@ntu.edu.tw (C.-C.C.)

**Keywords:** hexagonal photonic crystal, sensor

## Abstract

The sensitivity of traditional diffraction grating sensors is limited by the spatial resolution of the measurement setup. Thus, a large space is required to improve sensor performance. Here, we demonstrate a compact hexagonal photonic crystal (PhC) optical sensor with high sensitivity. PhCs are able to diffract optical beams to various angles in azimuthal space. The critical wavelength that satisfies the phase matching or becomes evanescent was used to benchmark the refractive index of a target analyte applied on a PhC sensor. Using a glucose solution as an example, our sensor demonstrated very high sensitivity and a low limit of detection. This shows that the diffraction mechanism of hexagonal photonic crystals can be used for sensors when compact size is a concern.

## 1. Introduction

Optical sensors have attracted a great deal of attention because they offer several advantages, such as non-invasive measurement, high specification, high sensitivity, real-time monitoring, and low cost, compared to other conventional analytical techniques [1]. Among various types of optical biosensors, diffraction gratings can specifically or nonspecifically interact with target materials. Diffracted beams are measured by correlating the change in optical properties to the geometrical parameters of the gratings and the refractive index of the target material. For example, the wavelength shift, spatial distance shift, and diffraction power efficiency of the diffraction patterns can be monitored to detect types or even concentrations of the target. However, the performance of traditional diffraction grating sensors is strongly dependent on spatial resolution. A large space is needed to obtain high sensitivity. The periodic patterns of diffraction based sensors can also be implemented by photonic crystals (PhCs), in which light interacts with the periodic structure.

PhC represents an intriguing solution to achieve high sensing performance. Sensors employing photonic architectures include ring resonators [1], surface plasmon resonance (SPR) sensors [2], microdisks [3], microspheres [4] and guided mode resonance (GMR) [5,6] sensors. For example, based on GMR theory, PhC sensors can be used to detect immunoglobulinG antibodies [7], serve as a grating coupler in SPR systems to monitor biomolecular interactions [8] and function as a gas sensor [9]. Since PhCs can confine light to a very small volume, chemical species can be characterized by nanometer dimensions [10]. In addition, PhC sensors can be integrated with microfluidic systems, endowing compact sensor chips with advanced chemical surface functionalization techniques [11]. 

In this work, with the purpose of designing a miniature optical sensor for a handheld device [12] and IoT (Internet of Things) sensing [13], we fabricated 2-dimensional (2D) hexagonal PhCs on a Si substrate. The diffracted beams from the PhCs distributed at various azimuthal angles. They satisfied phase matching conditions in terms of optical wavelength, PhC period and material applied on the surface. However, certain wavelengths of the incident beam became evanescent after diffraction because they failed to satisfy phase matching. Based on the unique diffraction properties of hexagonal PhCs, we benchmarked the sensitivity of the device according to refractive index change, using glucose as the target analyte. By analyzing the spectrum of the diffracted beam, the onset of the wavelength at which diffracted beams became evanescent was employed to correlate the refractive index of the glucose solutions. Our sensor showed high sensitivity, while avoiding the problem of spatial resolution compared to traditional grating sensors.

## 2. Materials and Methods

### 2.1. Design and Fabrication of the Hexagonal 2D Photonic Crystal

**Figure 1:**
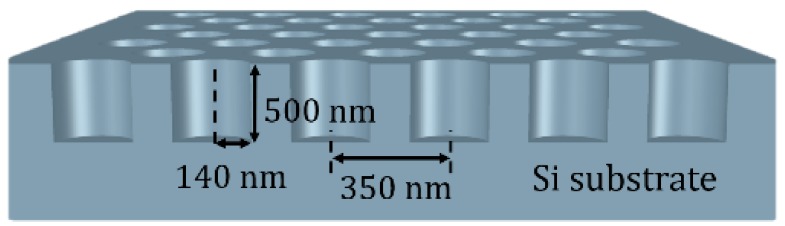
Schematic diagram of the 2D photonic crystal (PhC) sensor. The geometry of the nanohole arrays is labeled.

**Figure 2:**
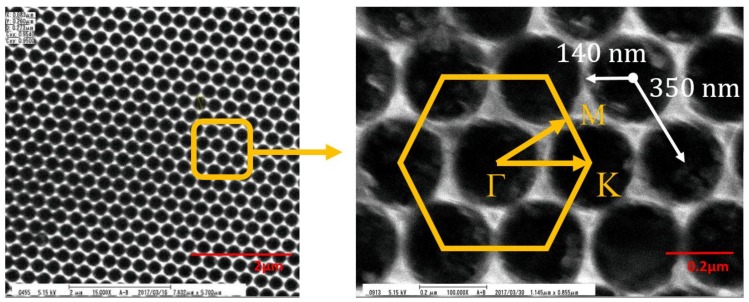
SEM images of PhCs.

The PhC sensor was fabricated on a Si substrate. The process started from first depositing a 250 nm-thick layer by Plasma Enhanced Chemical Vapor Deposition (PECVD) as the hard mask. The hexagonal 2D PhCs (nanohole arrays) were patterned by electron beam lithography. The patterned hard mask enabled the precise transfer of the PhC pattern to the Si substrate by Reactive-Ion Etching (RIE). The dielectric was then removed by buffer oxide etchant (BOE) wet etching. A schematic diagram of the PhC pattern is shown in Figure 1. The PhC nanohole arrays were hexagonally arranged, with a period (a) of 350 nm, radius (r) of 140 nm, and depth of 500 nm. The geometry of the PhC was designed so that the diffraction spectrum was in the visible wavelength. The size that the nanohole PhC pattern covered was around 300 × 300 μm^2^. The scanning electron micrograph (SEM) images are shown in Figure 2.

### 2.2. Measurement Setup

**Figure 3:**
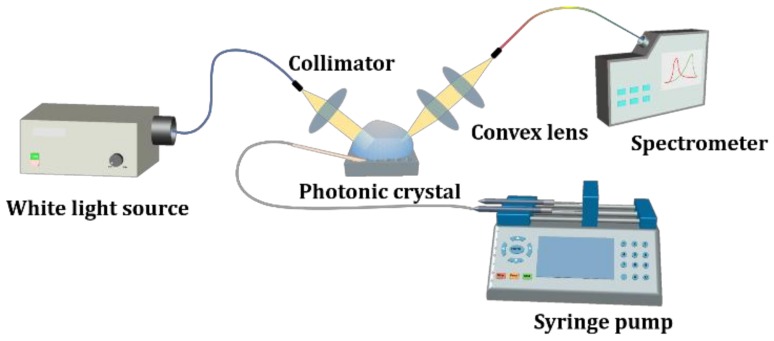
Experimental setup of the optical characterizations of the PhC arrays and detection of the refractive index of the glucose solution. The target glucose solution was injected by a syringe pump into the PhC surface.

There were two steps of measurement in this work. We first characterized the light diffraction behavior of the PhC structure, then performed glucose sensing. In the first part, to analyze the diffraction modes of the hexagonal lattices, broadband white light emission from a Xenon lamp was coupled into a multi-mode optical fiber and then was collimated before shining on the PhC surface. The incident plane of the light was chosen to be aligned with the Γ-K direction, which corresponds to a grating period of 350 nm (see Figure 2). The spot size of the incident light was around 1.5 mm in diameter. Only light diffracted by the PhC could be observed at various azimuthal angles. The diffracted modes were analyzed by a spectrometer (Ocean Optics, Largo, FL, USA, HR4000). Second, using the PhC structure as a sensor, we correlated the refractive index with the diffraction spectra using a glucose solution. A syringe pump (Chemyx Inc., Stafford, TX, USA, Fusion 200) was employed to control the flow rate and volume of the glucose solution. A schematic diagram of our two-step measurement setup is shown in Figure 3.

## 3. Results and Discussion

### 3.1. Optical Bhavior of the Hexagonal PHC

**Figure 4:**
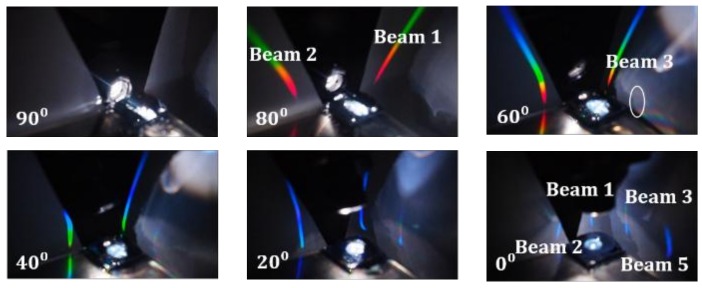
Images of diffracted beams under different incident angles, as labelled in the lower-left segment of each photo.

**Figure 5:**
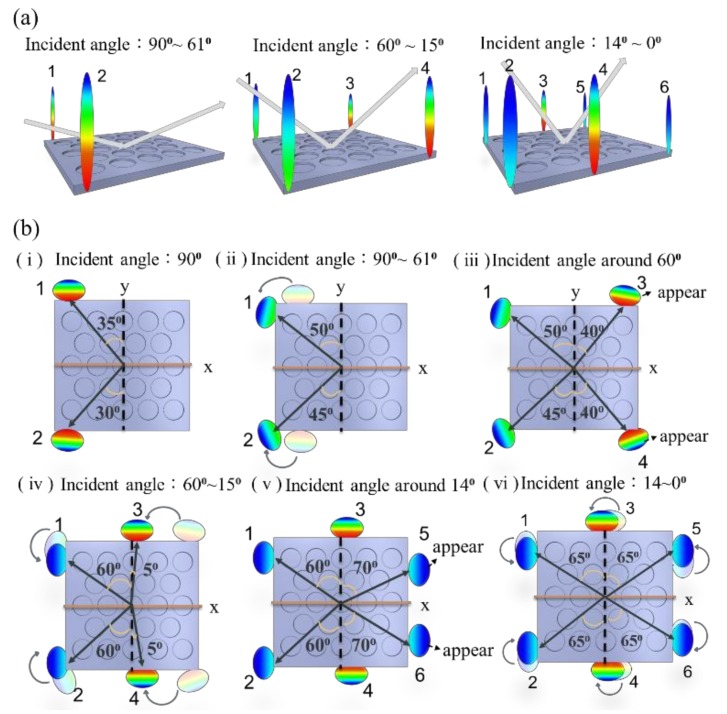
Illustrations of the diffracted beams that were deviated from the plane of incident light. The diffraction behaviors are drawn from(**a**) a side view and (**b**) a top view.

For periodic optical grating, the reflective modes fell on the same plane as the incident light. The 2D PhCs allowed incident light to be diffracted in various directions, as long as the conservation of momentum was satisfied. For the hexagonal PhC structure, the diffracted beams appeared at various azimuth angles above the PhC surface, in addition to the same incident plane. Figure 4 shows the diffracted patterns taken by a digital camera under white light illumination. The incident angle is marked at the bottom-left corner of each photo. Here, 90° is in the horizontal direction while 0° is from the surface normal of the sample. With the increase in incident angle from 0°, the number of diffracted beams decreased from six to two. To help visualize the diffraction behavior, schematic diagrams of the diffracted beams are drawn in Figure 5. Here, we only show optical beams that are not aligned with the incident light plane. When the incident angle was between 90° and 61°, two beams (beam 1 and beam 2) existed that were not in the same incident plane (see Figure 5a). As we tilted the incident light from 90° toward the surface normal of the device, beam 1 and 2 gradually moved to the direction of the incident light plane (see Figure 5b(ii)). At an incident angle below 60°, two additional beams, beam 3 and 4, rose above the PhC surface at a location 50° away from the *x*-axis, as shown in Figure 5b(iii). When we kept shrinking the incident angle, the diffracted beams had the tendency to move toward the incident plane, making room for additional beams to appear. When the incident angle was between 0° and 14°, there were a total of six diffracted beams that fell away from the plane of incident light.

**Figure 6:**
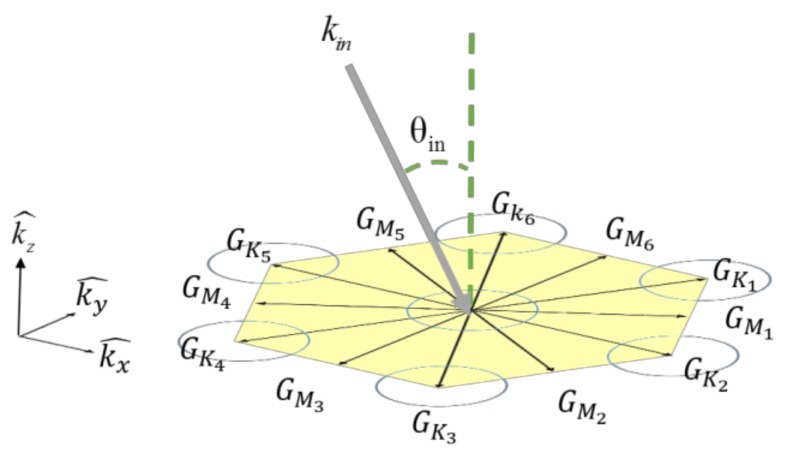
Schematic diagram of the reciprocal space of the 2D hexagonal PhC. The incident beam $\;k_{in}$ interacted with the lattice vectors and was diffracted in six possible directions in the azimuthal space.

The optical behavior of our 2D-PhC can be explained based on the phase matching equation given by the conservation of crystalline momentum:

(1) $$k_{in}+mG_{x,y}=k_{out},k_{in}=\frac{2\pi n}{\lambda }\sin\theta_{in}$$

where $k_{in}$ is the incident in-plane wavevector; λ is the wavelength of incident light; $\theta_{in}$ is the angle between the surface normal and incident light; $k_{out}$ is the in-plane wavevector of the diffracted beam; *m* is the harmonic order of diffraction; *n* is refractive index of the dielectric; and *G* is the reciprocal lattice vector given by PhCs. Light can be extracted from PhCs and radiated into the air, which is attributed to the coupling of the in-plane wavevector and the reciprocal lattice vector *G_x,y_*. Because of the symmetry of the lattice structure, the direction of the primitive cells in reciprocal space were a key factor of concern. Figure 6 shows the reciprocal lattice of hexagonal PhCs. In Figure 6, six reciprocal lattice vectors (from G_k1_ to G_k6_) existed in the first Brillouin zone [14]. As the harmonic order of diffraction (i.e., *m*) increased, the number of lattice vectors (from G_m1_ to G_m6_) grew larger. When the phase-matching condition was fulfilled, the guided light could be diffracted into the air. In our measurement, as the incident angle ($\theta_{in}$) titled toward the normal direction, the in-plane vector ($k_{in}$) in Equation (1) was smaller so that more reciprocal lattice vectors satisfied the equation. The number of diffracted beams appearing in space increased. Due to the hexagonal PhC pattern arrangement, at a small incident angle (below 0° in our case), the number of diffracted beams was six.

**Figure 7:**
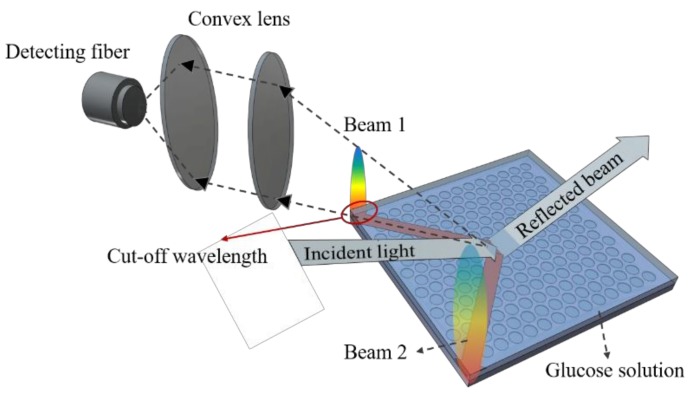
Illustration of diffracted beam detection. The optical beam was collimated by the focal lense before it was collected by a fiber. The spectrum of the optical beam was analyzed by a spectrometer.

Equation (1) also implies that diffraction behavior is dependent on the refractive index of the material applied on the PhC sensor surface. All of the diffraction patterns that were visible on top of the sensor surface met the phase matching conditions. As the wavelength of the incident light increased to a certain value where the corresponding $k_{in}$ wavevector could not satisfy Equation (1), the diffracted beam became an evanescent wave (imaginary number of the wavevector). We defined a cut-off wavelength as a wavelength in the boundary between the solution of real and imaginary numbers (see Figure 7). Since $k_{in}$ is proportional to the refractive index (*n*), the cut-off wavelength is correlated to the refractive index. When the incident angle or refractive index increases, the cut-off wavelength will shift to a longer wavelength. Traditional optical biosensors characterize the refractive index of target materials by measuring the angular shift or wavelength shift of the reflected light. Spatial resolution has a tremendous influence on sensitivity. It means that the longer the distance between the reflected light and detecting instrument, the higher the sensitivity is. The cut-off wavelength approach in our hexagonal PhC avoided the limits of spatial resolution. We were able to detect the refractive index change by determining the cut-off condition from the spectra of the diffracted beams.

### 3.2. Principle of PhC Sensing

Figure 7 elucidates the principle of detection using a PhC sensor. The PhC structure scatters the incident optical wave to different diffracted modes, including a zero-order reflected mode and other diffracted beams. We analyzed the cut-off wavelength from the diffracted spectrum of one beam by collecting optical power with confocal lenses. For wavelengths longer than the cut-off, since the diffracted light became evanescent, we observed a roll-over at longer wavelengths in the diffracted spectrum. Diffraction is modulated when a target solution is introduced on the surface of a PhC sensor. Thus, the cut-off wavelength is dependent on the refractive index of the target solution.

### 3.3. Cut-Off Wavelength Measurement and Data Acquisition

**Figure 8:**
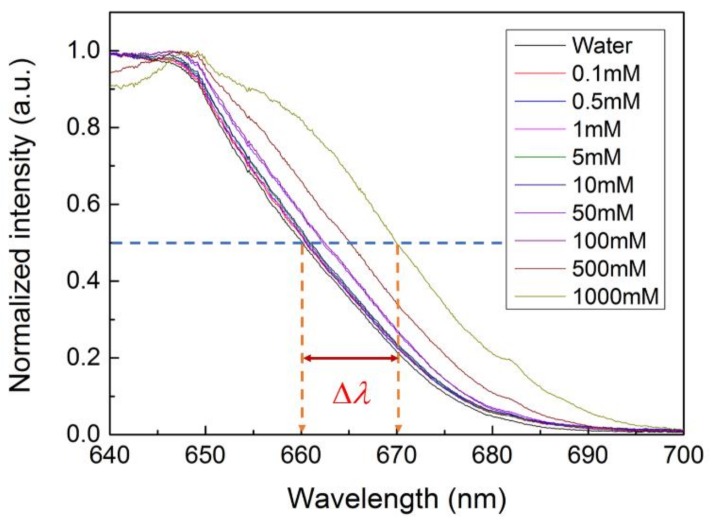
Diffraction spectra from the PhC sensor with the glucose solutions applied. The cut-off wavelength shift, Δλ, indicated the capability of detecting the refractive index difference of target analytes with a PhC sensor. Even though the diffraction spectrum covered the visible wavelength range, we only collected longer wavelength (640~700 nm) results, where cut-off wavelength could be determined.

We explored the detection capabilities of the hexagonal PhC structure using a glucose solution. As shown in Figure 3, the glucose solution was injected to the PhC surface by a syringe pump. We characterized the diffraction behaviors of glucose solutions with concentrations ranging from 0.1 to 1000 mM. Among the diffraction patterns at various incident angles, the diffraction of only two beams possessed the highest light intensity. Thus, we set the incident angle of 70° for our sensing experiment and diffracted beam 1 in Figure 5b(ii) was analyzed. The cut-off wavelength, defined as the wavelength at half of the maximum intensity from the spectra in Figure 8, was employed to benchmark the refractive index. When the glucose concentration increased, the refractive index increased, resulting in a red shift of the diffraction spectrum.

### 3.4. Detection Limit and Sensitivity

**Figure 9:**
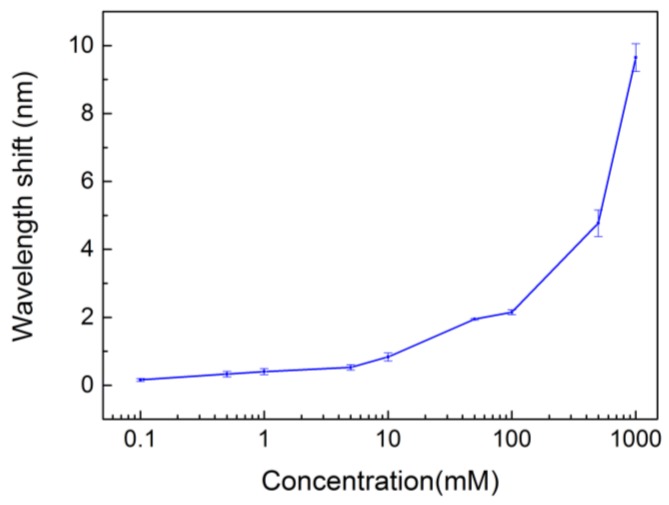
Cut-off wavelength of glucose solutions with different concentrations.

The cut-off wavelength shifts of water and glucose solutions are shown in Figure 9. In this experiment, six PhC devices were tested for each glucose concentration. The average wavelength shift for 0.1 mM was 0.161, which was around four times the standard deviation, 0.044. The detection limit of our sensor was about 0.1 mM, which was much lower than conventional blood glucose detectors for Diabetes mellitus (DM) patients [15].

**Figure 10:**
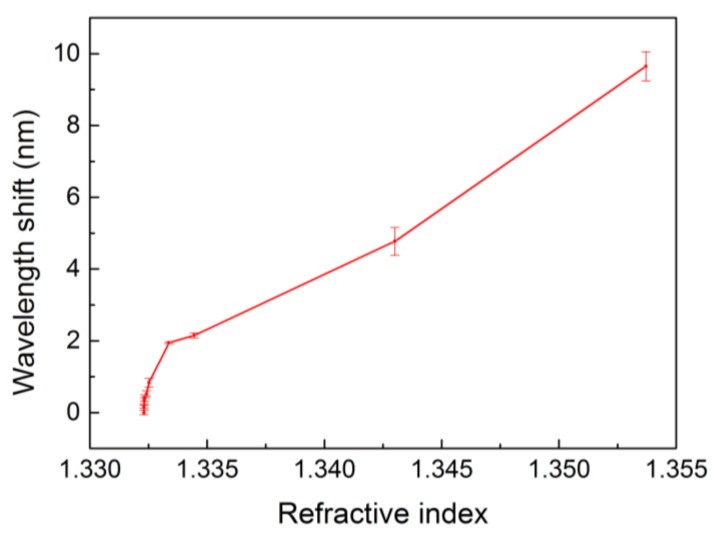
Cut-off wavelength shift vs. refractive index of water and glucose solutions with concentration ranging from 0.1 to 1000 mM.

The optical detection properties of the sensor could be quantitatively estimated by the sensitivity parameter, $S=\frac{\Delta \lambda }{\Delta n}$, which is defined as the ratio of wavelength shift (Δλ) to refractive index change (Δn). We converted the glucose concentration to the corresponding refractive index. Figure 10 shows the correlation between the cut-off wavelength shift and the refractive index. For the glucose solution above 10 mM, the slope in Figure 10 was calculated to be 378 nm/RIU. As for the concentration below 10 mM, the slope was 3091 nm/RIU, which suggests the PhC sensors are capable of high sensitivity.

## 4. Conclusions

We demonstrated the evanescent properties of optical diffraction using a 2D hexagonal PhC structure. The device can be used for sensor applications by correlating the refractive index with the measured cut-off wavelength. For glucose solution above 10 mM, the sensitivity was around 378 nm/RIU, while a much higher sensitivity of 3091 nm/RIU was achieved for concentrations below 10 mM. The limit of detection was 0.1 mM. The sensitivity of our PhC sensors was compatible or even superior to that of other PhC-based sensors. The advantage of the cut-off wavelength approach is that large spaces of measurement setup can be avoided in the high-resolution sensing of typical grating sensors.
